# Toxicogenetic analysis of Δ9-THC-metabolizing enzymes

**DOI:** 10.1007/s00414-020-02380-3

**Published:** 2020-07-25

**Authors:** Angela Gasse, Marielle Vennemann, Helga Köhler, Jennifer Schürenkamp

**Affiliations:** Institute of Legal Medicine, University Hospital Münster, Röntgenstr. 23, 48149 Münster, Germany

**Keywords:** Toxicokinetic, Cannabis, Δ9-THC metabolism, *phase I* enzymes, CYP2C9, CYP2C19

## Abstract

While the impact of genetic polymorphisms on the metabolism of various pharmaceuticals is well known, more data are needed to better understand the specific influence of pharmacogenetics on the metabolism of delta 9-tetrahydocannabinol (Δ9-THC). Therefore, the aim of the study was to analyze the potential impact of variations in genes coding for *phase I* enzymes of the Δ9-THC metabolism. First, a multiplex assay for genotyping different variants of genes coding for *phase I* enzymes was developed and applied to 66 Δ9-THC-positive blood samples obtained in cases of driving under the influence of drugs (DUID). Genetic and demographic data as well as plasma concentrations of Δ9-THC, 11-hydroxy-Δ9-tetrahydrocannabinol (11-OH-Δ9-THC), and 11-nor-9-carboxy-Δ9-THC (Δ9-THC-COOH) were combined and statistically investigated. For cytochrome P450 2C19 (CYP2C19) variants, no differences in analyzed cannabinoid concentrations were found. There were also no differences in the concentrations of Δ9-THC and 11-OH-Δ9-THC for the different allelic CPY2C9 status. We recognized significantly lower Δ9-THC-COOH concentrations for CYP2C9*3 (*p* = 0.001) and a trend of lower Δ9-THC-COOH concentrations for CYP2C9*2 which did not reach statistical significance (*p* = 0.068). In addition, this study showed significantly higher values in the ratio of Δ9-THC/Δ9-THC-COOH for the carriers of the CYP2C9 variants CYP2C9*2 and CYP2C9*3 compared with the carriers of the corresponding wild-type alleles. Therefore, an impact of variations of the *CYP2C9 gene* on the interpretation of cannabinoid plasma concentrations in DUID cases should be considered.

**Electronic supplementary material:**

The online version of this article (10.1007/s00414-020-02380-3) contains supplementary material, which is available to authorized users.

## Introduction

**Fig. 1:**
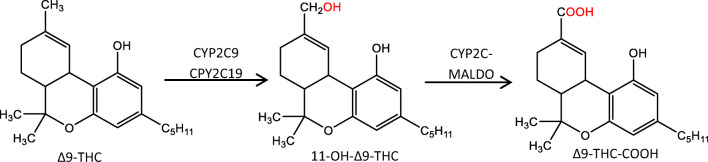
Main *phase I metabolism* of Δ9-THC

The knowledge about pharmacogenetic and its meaning for the optimum drug dosage or the minimization of dose-related adverse drug reactions has increased constantly over the last years [1, 2]. With so-called personalized medicine, a gradual integration of this knowledge into clinical practice takes place [3–6]. In comparison, the transfer to the field of forensic toxicology is still at the very beginning. However, there are a number of case reports and review articles aiming at investigating and evaluating the benefit of toxicogenetic analyses for forensic issues [7–14]. In summary, they conclude that in single cases, genotyping of the relevant metabolizing enzymes can be a further tool for a comprehensive interpretation of analytical results. In this context, genetic variations of genes coding for Δ9-THC-metabolizing enzymes are of high relevance but very little studied, and their impact on plasma concentrations of Δ9-THC and its metabolites is not sufficiently understood. Highly polymorphic enzymes are described for Δ9-THC metabolism (see Fig. 1): In *phase I*, the majority of Δ9-THC is hydroxylated to 11-OH-Δ9-THC via CYP2C9 and CYP2C19 [15]. An alternative minor route is Δ9-THC metabolism to 8β-OH-Δ9-THC and 9,10-epoxyhexahydrocannabinol via CYP3A4 [16, 17]. 11-OH-Δ9-THC is further oxidized to the psychoinactive Δ9-THC-COOH. This step is catalyzed by a microsomal aldehyde oxygenase (MALDO) whose affiliation to the CYP2C subfamily is discussed [16].

Due to numerous in vitro and in vivo studies with different substrates, the impact of genetic variations in *CYP2C9* and *CYP2C19* genes on the activities of the corresponding enzymes is well described, and the functionally most relevant ones are summarized in Table 1.

**Table 1:** Allele frequencies of CYP2C9 and CYP2C19 polymorphisms in Caucasians and their effect on the enzyme activity [18]

Polymorphism	Allele frequency [%]	Enzyme activity
CYP2C9*2 430 C>T	14.0	Decreased
CYP2C9*3 1075 A>C	6.4	Decreased
CYP2C19*2 681 G>A	14.5	None
CYP2C19*3 636 G>A	< 1	None
CYP2C19*17-806 C>T	18.8	Increased

Concerning the metabolism of Δ9-THC, the decrease in enzyme activity in the conversion from Δ9-THC to 11-OH-Δ9-THC has already been described in vitro for the variations CYP2C9*2 and CYP2C9*3 with a reduction of the intrinsic clearance of 70% [19]. Looking at the few in vivo results [20, 21] especially the work of Sachse-Seeboth et al. [21] revealed the CYP2C9*3 variant as a significant determinant of Δ9-THC and Δ9-THC-COOH kinetics. After oral administration of Δ9-THC to 43 volunteers, they observed a median area under the curve of Δ9-THC which was threefold higher in CYP2C9*3/*3 homozygotes compared with CYP2C9*1/*1 homozygotes and that of Δ9-THC-COOH which was 70% lower in CYP2C9*3/*3 homozygotes compared with CYP2C9*1/*1 homozygotes. However, due to the absence of sufficient data particularly regarding the most common form of cannabis consumption, which is assumed to be smoking, the role of genetic variations in Δ9-THC-metabolizing enzymes and their impact on the interpretation of analytical results in forensic toxicology have not yet been fully clarified. Therefore, the aims of this study were (1) the development of genotyping methods for variants of genes encoding enzymes of the Δ9-THC *phase I* metabolism, (2) the application of these methods to DUID case blood samples tested positive for cannabinoids, and (3) the comprehensive statistical investigation of the impact genetic variations might have on forensic analytical results.

## Material and methods

### Samples

A total of 66 participants were recruited among persons who had to undergo a driving aptitude assessment test at the Institute of Legal Medicine in Münster because of driving under the influence of cannabis. After written informed consent has been obtained from each volunteer, blood was taken for DNA analysis of genes coding for Δ9-THC-metabolizing enzymes. Additionally, data on plasma concentration from the DUID cases, age, gender, body height, weight, biogeographic origin, and consumption pattern concerning cannabis, nicotine, alcohol, and medication were collected. The study was approved by the local ethical committee.

### Chemical toxicological analysis

Qualitative and quantitative analyses of Δ9-THC, 11-OH-Δ9-THC, and Δ9-THC-COOH in plasma samples of DUID cases were carried out with a validated method using gas chromatography-mass spectrometry (GC-MS) following solid-phase extraction. Briefly, 1 ml plasma was diluted with 2 ml phosphate buffer (0.15 M, pH 6.0; potassium dihydrogen phosphate and sodium hydrogen phosphate, Merck, Darmstadt, Germany), and internal standard (5 ng Δ9-THC-d_3_, 5 ng 11-OH-Δ9-THC-d_3_, and 50 ng Δ9-THC-COOH-d_9_, Sigma-Aldrich, Steinheim, Germany) was added. After mixing (vortex) and centrifugation (2576×*g*, 10 min), the resulting supernatant was decanted to conditioned Chromabond® C18 columns (Macherey-Nagel, Düren, Germany). The columns were successively washed with 3 ml aqua bidest (AppliChem, Darmstadt), 1 mL 0.25 M acetic acid (Merck, Darmstadt, Germany), and 3 ml methanol/aqua bidest (50:50, v/v, Sigma-Aldrich, Steinheim, Germany: AppliChem, Darmstadt, Germany) followed by a drying step of 5 min under vacuum. After addition of 100 μl acetone (Sigma-Aldrich, Steinheim, Germany), the columns were again dried under vacuum for 20 min. The eluate of 1.5 ml acetone was evaporated to dryness at 40 °C in a stream of nitrogen. The extracts were derivatized using 30 μl MSTFA (N-methyl-N-(trimethylsilyl) trifluoroacetamide, Macherey-Nagel, Düren, Germany) at 70 °C for 20 min. GC-MS analysis was performed using an Agilent Technologies 6890 N system (Agilent Technologies, Waldbronn, Germany) equipped with a mass selective detector (5973) and a Combi PAL Autosampler system (CTC analytics, Zwingen, Switzerland). Mass selective detector was operating in electron impact (EI) selective ion monitoring (SIM) mode. GC separation was carried out with a capillary column OPTIMA® 5 MS Accent (30 m × 0.25 i.d. × 0.25 μm from Macherey-Nagel, Düren, Germany) with the following temperature program: 1.5 min at 150 °C, 9°/min up to 260 °C, hold for 6 min, 30°/min up to 300 °C, hold 8 min. The helium gas flow rate was 1.0 ml/min. The temperatures of the injector, the transfer line, the ion source, and the quadrupole were 250 °C, 280 °C, 230 °C, and 150 °C respectively. The injection volume was 2 μl. The m/z values used for identification and quantification of the trimethylsilyl derivatives in the SIM-mode were as follows (target ion underlined): Δ9-THC 371, 386, 303; Δ9-THC-d_3_ 374, 389; OH-Δ9-THC 371, 459, 474; OH-Δ9-THC-d_3_ 374, 462; Δ9-THC-COOH 371, 297, 473; Δ9-THC-COOH-d_9_ 380, 303. Validation was carried out according to the criteria of the Society of Toxicological and Forensic Chemistry (GTFCh) guidelines [22]. Briefly, the limit of detection (LOD) was 0.2 ng/ml for Δ9-THC/11-OH-Δ9-THC and 1.1 ng/ml for Δ9-THC-COOH. The limit of quantification (LOQ) was 0.5 ng/ml for Δ9-THC/11-OH-Δ9-THC and 3.2 ng/ml for Δ9-THC-COOH. Linearity was shown from 0.5 to 10 ng/ml for Δ9-THC/11-OH-Δ9-THC and from 5 to 100 ng/ml for Δ9-THC-COOH, respectively. Samples above the calibration range were measured again after dilution. The bias of low (Δ9-THC 2.0 ng/ml; 11-OH-Δ9-THC 2.1 ng/ml; Δ9-THC-COOH 8 ng/ml) and high (Δ9-THC 8.0 ng/ml; /11-OH-Δ9-THC 8.0 ng/ml; Δ9-THC-COOH 72.5 ng/ml) quality control level at eight different days was below 10%, except for Δ9-THC-OH (−15.9%), and relative standard deviation was below 10% for all analytes. Selectivity was checked by extracting six different lots of human blank plasma, two spiked blank plasma samples with deuterated internal standards, and two blank plasma samples spiked with 14 drugs expected in forensic samples (amphetamines, opiates, cocaine, and their metabolites, respectively) at higher concentrations. In principle, THC-COO-glucuronide can be cleaved to THC-COOH before analytical detection, e.g., during extraction, derivatization, or ionization. This would result in incorrectly high THC-COOH concentrations. Therefore, seven spiked plasma samples with a high THC-COO-glucuronide concentration (200 ng/ml) were examined with regard to the formation of THC-COOH. The hydrolysis rate was calculated as the amount of THC-COO-glucuronide which was converted to THC-COOH and amounts to 3.15%. Recovery for all analytes was above 75%.

### DNA analysis

#### DNA extraction

Cotton swabs soaked with blood were air dried and stored at room temperature. Approximately 0.3 cm^2^ was cut from each swab, covered with 500 μl HPLC-water, and incubated 15 min at room temperature. After centrifugation, the aqueous supernatant was discarded, and the swab was covered with 200 μl of 5% Chelex (BioRad, Munich, Germany) and 5 μl of 20 mg/ml proteinase K and incubated at 56 °C for 60 min and finally heated to 100 °C for 8 min [23]. DNA extracts were stored at − 20 °C until further use.

#### Marker selection and assay design

We selected five single nucleotide polymorphisms (SNPs) influencing expression rate or enzyme activity of the *phase* I metabolism enzymes CYP2C9 and CYP2C19 (see Supplement 1). Five amplification primer sets were designed using Primer3 software [24, 25] and purchased from Biomers (Ulm, Germany). For application in a multiplex reaction, the primers were designed with comparable annealing temperatures and a length between 18 and 30 nucleotides, a G/C content of 40–60%, and a maximum sequence of four identical bases [26]. In order to avoid unwanted amplification products by primer hybridization to related sequences, the specific binding to the target sequence was checked using the BLAST search module [27]. The hybridization of the primers among each other and the formation of hairpin structures within a primer sequence were examined using the software AutoDimer [28]. One minisequencing primer for each SNP was designed so that its 3′-end was located one base upstream of the SNP position. To enable separation of sequencing products by fragment lengths analysis, a poly-T-tail was added to the 5′-end of the primers to obtain fragment lengths between 30 and 50 nucleotides.

Primers were initially checked in singleplex reactions. Polymerase chain reaction (PCR) was performed using 6.1 μl HPLC-grade water, 1.5 μl MgCl_2_ (25 mM), 0.2 μl of 20 mg/ml bovine serum albumin (BSA, MoBiTec, Göttingen, Germany), 0.1 μl AmpliTaq Gold polymerase (5 U/ml), 1.2 μl AmpliTaq Gold buffer, 1.2 μl of 10 mM dNTPs (all Thermo Fisher Scientific, Darmstadt, Germany), 0.1 μl of forward and reverse primer (10 pmol/μl), and 2 μl of DNA extract. A T3 Thermocycler (Biometra, Göttingen, Germany) was used with 15 min 94 °C followed by 30 cycles of 93 °C, 58 °C, and 72 °C for 30 s each and a final elongation step with 72 °C for 30 min. Amplification success was confirmed by polyacrylamide gel electrophoresis (PAGE, 9.3%). Specificity of primer binding and identity of amplicon sequences were verified by Sanger sequencing using Big Dye Terminator v1.1 Cycle Sequencing Kit, Applied Biosystems [29].

#### Multiplex assay

The multiplex amplification method was adopted as described for the singleplex PCR. Successful amplification was verified by PAGE. For removal of unbound nucleotides and excess primers, the PCR products were treated with 0.04 μl exonuclease I (20 U/μl, New England Biolabs, Frankfurt, Germany) and 1.25 μl shrimp alkaline phosphatase (SAP; 1 U/μl) (Roche, Mannheim, Germany) for 75 min at 37 °C followed by denaturation at 78 °C for 15 min [26].

Minisequencing (single base extension, SBE) was performed using the SNaPshot Multiplex kit (Thermo Fisher Scientific) with 0.5 μl HPLC-water, 1 μl amplification product, 0.5 μl of each minisequencing primer (1 pmol/μl for SNP-2C9*2 and 10 pmol/μl for SNP-2C9*3, -2C19*2, -2C19*3, -2C19*17), and 1 μl Ready Reaction SNaPshot Mix. A T3 Thermocycler (Biometra, Göttingen, Germany) was used with 25 cycles of 96 °C for 10 s, 55 °C for 5 s, 60 °C for 30 s, and a final extension step with 60 °C for 10 min. Sequencing products were treated with SAP for 95 min at 37 °C followed by denaturation at 78 °C for 15 min.

Fragment length analysis was performed by capillary electrophoresis on a 3130 Genetic Analyzer using polymer POP7 (Performance Optimized Polymers7) and internal lane standard Liz120 (all Thermo Fisher Scientific) following manufacturer’s recommendations for SNaPshot product analysis. Allele assignment was done with the software GeneMapper ID Version 3.2.1. An allelic ladder was produced by using different samples in combination to represent each of the 10 possible alleles, which were previously identified by Sanger sequencing. Since none of the samples showed the variation CYP2C19*3, only the wild-type expression could be included in the ladder. The assay’s sensitivity was tested using a twofold serial dilution of a known DNA sample ranging from 2 ng to 7.8 pg. In addition, a 10 ng DNA sample was used to investigate the robustness of the assay against extremely high amounts of DNA input.

#### Statistical analysis

The binomial test was used to investigate the allele frequencies within the study group compared with the allele frequencies in the literature among Caucasians [30]. When combining genetic data and plasma concentrations, the differences between two groups were investigated using the Mann-Whitney *U* test (*α* = 5%) in SPSS (version 22.0). For the distribution-independent comparison of more than two groups, the nonparametric Jonckheere-Terpstra trend test was also used in SPSS. Here, the presence of a trend between the genotypes was tested for the following, previously defined group sequence: CYP2C9 *1/*1, *1/*2, *2/*2, *1/*3, *2/*3.

## Results

### DNA method development

**Fig. 2:**
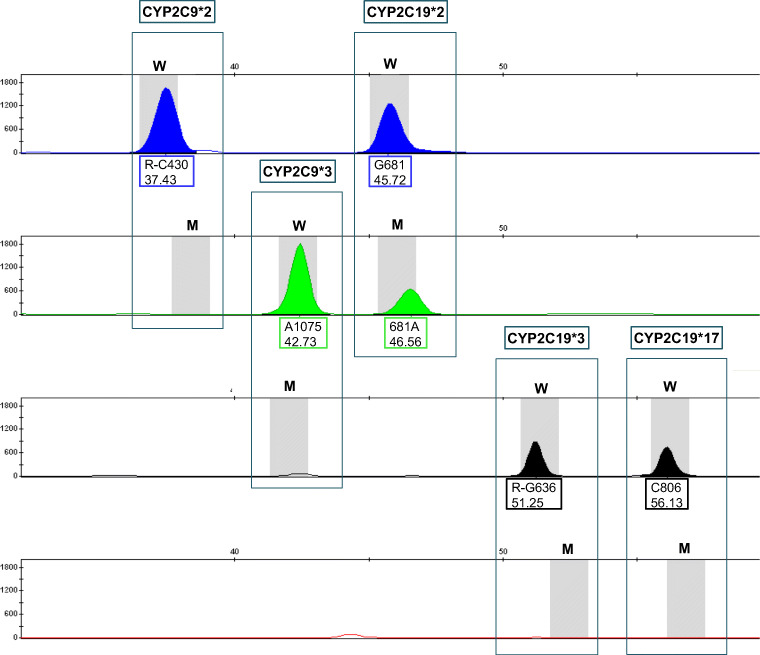
Electropherogram of SNaPshot products of a multiplex reaction. The horizontal scale shows fragment length in nucleotides (nt). The vertical scale shows signal intensities in relative fluorescence units (RFU). Results are shown in four color channels. The five boxes contain the five polymorphic DNA sites investigated with the two possible characteristics (gray bars; W = wild type, M = mutation). Signals indicate the detected alleles. The upper number in the labeling box of each signal corresponds to the position in the gene, with the letter before the number representing the base of the wild type and the letter after the number the base of the mutation. The lower number corresponds to the measured fragment length in nucleotides

The multiplex minisequencing assay for the simultaneous detection of five SNPs was validated successfully. The developed method allows the simultaneous detection of the two and three relevant SNPs on the CYP2C9 and CYP2C19 gene. After capillary electrophoretic separation of the multiplex minisequencing products, the retention order of the SNaPshot products in the electropherogram was corresponding to the primer lengths as follows: CYP2C9*2 - CYP2C9*3 - CYP2C19*2 - CYP2C19*3 - CYP2C19*17. An example profile is given in Fig. 2. It shows the homozygous wild type for CYP2C9*2, CYP2C9*3, CYP2C19*3, and CYP2C19*17 and the heterozygous mutation for CYP2C19*2.

### Study results

A total of 66 blood samples were analyzed genetically by a newly developed multiplex SNaPshot method targeting CYP2C9 and CYP2C19 genotypes. Additionally, the plasma of all samples was analyzed by a routinely used GC-MS method for quantification of cannabinoids. The range of the cannabinoid concentrations for all samples was 0.7–26.8 ng/ml for Δ9-THC, n.d.–8.3 ng/ml for 11-OH-Δ9-THC, and 8.6–149 ng/ml for Δ9-THC-COOH (Supplement 2).

#### Participants

Demographic data (age, body height, weight, and consumption pattern concerning cannabis, nicotine, alcohol, and medications; see Supplement 3) did not differ significantly between the CYP2C9 and CYP2C19 genotypes and had no significant impact on the cannabinoid plasma concentrations. All subjects were male, fifty originated from Germany; three from Russia; two each from Kosovo, Turkey, and Poland; and one each from Africa, Italy, Kazakhstan, the USA, Portugal, Romania, and Syria. No medicines which are known to be CYP2C9 or CYP2C19 inhibitors or inductors were reported.

Concerning the allele frequencies, none of the investigated gene variations, except CYP2C9*3, showed a statistically significant difference between the allele frequencies within the investigated group of volunteers and published allele frequencies among Caucasians [18]. Variation CYP2C9*2 was shown in 10.6% (*n* = 14) of the investigated alleles and in 14% of the alleles among Caucasians, CYP2C9*3 in 10.6% (*n* = 14) and 6.4%, CYP2C19*2 in 18.2% (*n* = 24) and 14.5%, CYP2C19*3 in 0% (*n* = 0) and < 0.1%, and CYP2C19*17 in 19.7% (*n* = 26) and 18.8%, respectively. The significant difference in the variation CYP2C9*3 (*p* = 0.022) is probably due to the rather small group of subjects.

### Evaluation of cannabinoid plasma concentrations depending on the CYP2C9 alleles

**Fig. 3:**
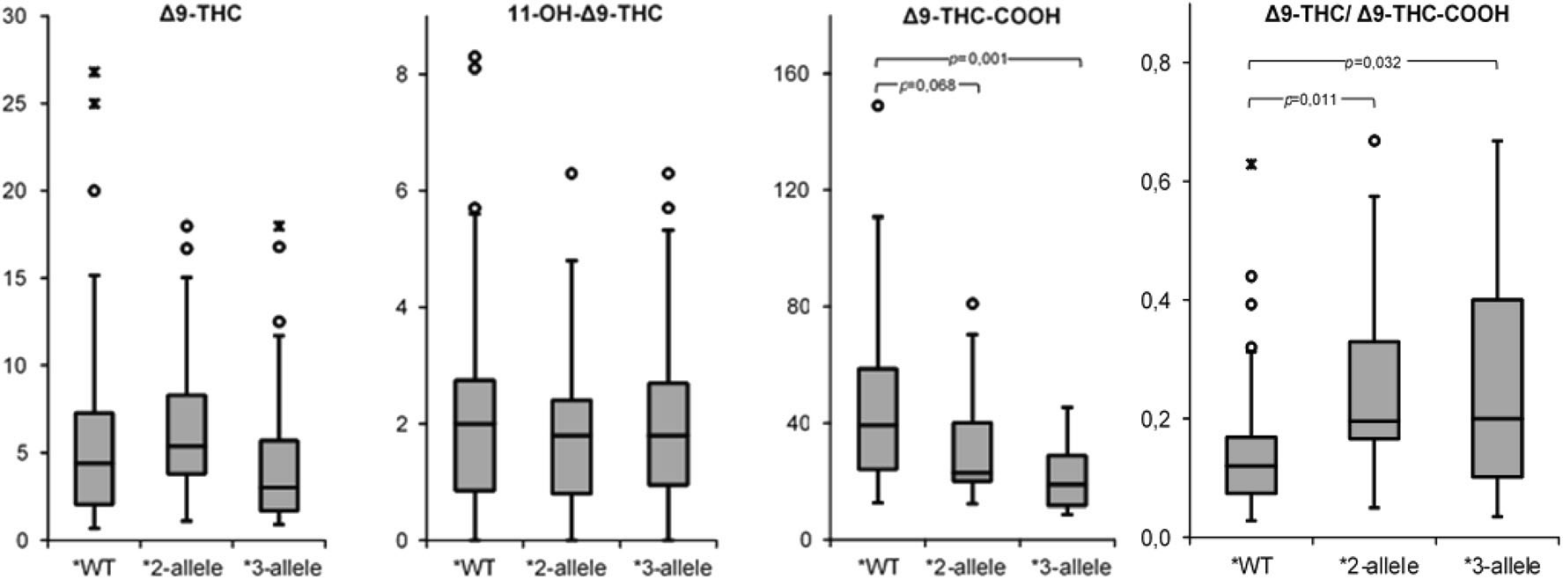
Box plot of plasma concentrations of THC (ng/ml), 11-OH-Δ9-THC (ng/ml), and Δ9-THC-COOH (ng/ml) as well as metabolic ratio of Δ9-THC (ng/ml)/Δ9-THC-COOH (ng/ml), for the carriers of the wild-type alleles (*WT, *n* = 43), the carriers of the CYP2C9*2 allele (*2 allele, *n* = 13), and the carriers of the CYP2C9*3 allele (*3 allele, *n* = 14). о = mild outliers, ӿ = extreme outliers

Plasma concentrations of Δ9-THC, 11-OH-Δ9-THC, and Δ9-THC-COOH depending on the CYP2C9 allelic status are shown in Table 2. A statistically significant difference was observed between the metabolic ratio (Δ9-THC ng/ml/Δ9-THC-COOH ng/ml) for CYP2C9*1/*1 and those for the two variations CYP2C9*2 and CYP2C9*3. The median of the metabolic ratios within the CYP2C9*2 group was 0.2, well above the median of the wild-type group of 0.12 (*p* = 0.011). The median of the metabolic ratios within the CYP2C9*3 group was also 0.2, well above the median within the wild-type group (*p* = 0.032). The results are graphically presented by means of boxplots in Fig. 3. Whereas the plasma concentration of Δ9-THC and 11-OH-Δ9-THC did not indicate any significant differences according the allelic status, the plasma concentrations of Δ9-THC-COOH did show a statistically significant difference (*p* = 0.001) between carriers of the CYP2C9 wild-type allele (median of 39.3 ng/ml) and CYP2C9*3 carriers (median of 18.9 ng/ml). Also, the CYP2C9*2 carriers showed considerably lower Δ9-THC-COOH concentrations compared with carriers of the CYP2C9 wild-type alleles with a median of 22.9 ng/ml. However, due to a *p* value > 0.05, statistical significance was not reached here, but a trend seems obvious (*p* = 0.068).

**Table 2:** Plasma concentrations as a function of the CYP2C9 alleles. The median values are given with the interquartile distances and the significance values

	CYP2C9				
	Wild-type	Allele*2		Allele*3	
*n*	43	13	*p*^a^	14	*p*^a^
Δ9-THC ng/ml	4.4 (2.1–7.3)	5.4 (3.8–8.3)	*n. s.*	3.0 (1.7–5.7)	*n. s.*
11-OH-Δ9-THC ng/ml	2.0 (0.9–2.8)	1.8 (0.8–2.4)	*n. s.*	1.8 (1.0–2.7)	*n. s.*
Δ9-THC-COOH ng/ml	39.3 (24.0–58.7)	22.9 (20.0–40.1)	*0.068*	18.9 (11.6–28.8)	*0.001*
[Δ9-THC ng/ml/Δ9-THC-COOH ng/ml]	0.12 (0.07–0.17)	0.20 (0.17–0.33)	*0.011*	0.20 (0.10–0.40)	*0.032*

^a^*p* values were determined by the Mann-Whitney *U* test and are derived by comparing the respective variation with the wild type; if the *p* value is below 0.05, the difference was considered statistically significant (*p* values between 0.05 and 0.1 are included to show possible trends)

*n. s.* not significant

### Evaluation of cannabinoid plasma concentrations depending on the CYP2C9 genotype

In the evaluation of plasma concentrations depending on the gene variant, no differentiation is made between individual genotypes. Therefore, an additional analysis of the different genotypes was performed. Concerning the genotype distribution, no statistically significant difference was observed between the frequencies within the investigated test population and those among Caucasians [31] except for genotype CYP2C9*2/*3. Genotype CYP2C9*1/*1 was shown in 65.2% (*n* = 43) of the investigated pool and in 65.3% among Caucasians, genotype CYP2C9*1/*2 in 12.0% (*n* = 8) and 20.4%, CYP2C9*2/*2 in 1.5% (*n* = 1) and 0.9%, CYP2C9*1/*3 in 15.2% (*n* = 10) and 11.6%, CYP2C9*2/*3 in 6.1% (*n* = 4) and 1.4%, and CYP2C9*3/*3 in 0% (*n* = 0) and 0.4%, respectively. The significant difference in the genotype CYP2C9*2/*3 (*p* = 0.002) is probably due to the rather small group of subjects. As the frequency of the homozygous CYP2C9*3/*3 genotype among Caucasians is round about 0.4%, it is not surprising that in our set of 66 persons, we had none of this genotype.

**Fig. 4:**
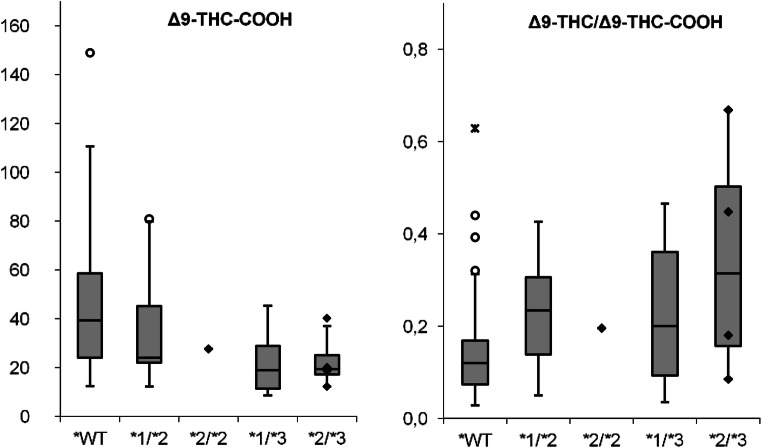
Box plot of plasma concentrations of Δ9-THC-COOH (ng/ml) and the metabolic ratio of Δ9-THC (ng/ml)/Δ9-THC-COOH (ng/ml) for the different CYP2C9 genotypes (WT *n* = 43; *1/*2 *n* = 8; *2/*2 *n* = 1; *1/*3 *n* = 10; *2/*3 *n* = 4). The individual values for the genotypes 2C9*2/*2 and 2C9*2/*3 are additionally represented in the form of diamonds due to the small group size. о = mild outliers, ӿ = extreme outliers

Following Sachse-Seeboth et al. [21], the presence of a trend in plasma concentrations in the following genotype order was investigated by means of a multigroup comparison: CYP2C9 *1/*1, *1/*2, *2/*2, *1/*3, *2/*3 (Table 3, Fig. 4). The multigroup comparison showed a significantly decreasing trend in the plasma concentrations of Δ9-THC-COOH (*p* = 0.001) and a rising trend in the metabolic ratios Δ9-THC/Δ9-THC-COOH (*p* = 0.007) for the investigated genotype order from CYP2C9*1/*1 to CYP2C9*2/*3. This corresponds well with previous observations regarding the influence of allelic status on cannabinoid concentrations described above. Concerning the plasma concentrations of Δ9-THC and 11-OH-Δ9-THC, again no difference between the genotypes could be detected.

**Table 3:** Multigroup comparison of CYP2C9 genotype and their cannabinoid concentrations

	CYP2C9					
	*1/*1	*1/*2	*2/*2	*1/*3	*2/*3	
*n*	43	8	1	10	4	*p*^a^
Δ9-THC ng/ml	4.4 (2.1–7.3)	5.8 (4.4–7.9)	5.4	3.0 (1.6–4.8)	7.4 (2.1–13.9)	*n. s.*
11-OH-Δ9-THC ng/ml	2.0 (0.9–2.8)	1.7 (0.6–2.3)	2.3	1.8 (1.1–2.3)	2.3 (0.8–4.1)	*n. s.*
Δ9-THC-COOH ng/ml	39.3 (24.0–58.7)	24.1 (22.0–45.2)	27.6	18.9 (11.3–28.8)	19.4 (17.1–25.1)	*0.001*
[Δ9-THC ng/ml/Δ9-THC-COOH ng/ml]	0.12 (0.07–0.17)	0.23 (0.14–0.31)	0.20	0.20 (0.09–0.36)	0.31 (0.16–0.50)	*0.007*

The median values are given with the interquartile distances and the significance values

^a^*p* values were determined by the Jonckheere-Terpstra trend test using a previously defined genotype order; at a *p* value of less than 0.05, the difference was considered statistically significant

*n. s.* not significant

### Evaluation of cannabinoid plasma concentrations depending on CYP2C19 variations

Since significant differences in cannabinoid concentrations between CYP2C9 genotypes were found, the CYP2C9 wild-type group (CYP2C9*1/*1) with *n* = 43 subjects was used as the baseline group to investigate the influence of the CYP2C19 variations. In this group, 16 carriers of CYP2C19 wild-type alleles were found, 13 carriers of the variation *2, and 17 carriers of the variation *17. Three persons were carriers of both the variation *2 and the variation *17, which were included in both groups, respectively. There was no carrier of the variant CYP2C19*3 in this group. Regarding the plasma concentrations, neither Δ9-THC nor its metabolites or the ratio Δ9-THC / Δ9-THC-COOH showed a significant difference depending on the investigated CYP2C19* alleles (Table 4).

**Table 4:** Plasma concentrations as a function of the CYP2C19 alleles. The median values are given with the interquartile distances and the significance values

	CYP2C19				
	Wild-type	Allele*2		Allele*17	
	*n* = 16	*n* = 13	*p*^a^	*n* = 17	*p*^a^
Δ9-THC ng/ml	4.4 (2.0–7.9)	3.4 (1.7–5.4)	*n. s.*	5.9 (3.0–7.9)	*n. s.*
11-OH-Δ9-THC ng/ml	2.1 (1.0–3.0)	1.8 (0.7–2.7)	*n. s.*	2.1 (1.0–3.0)	*n. s.*
Δ9-THC-COOH ng/ml	43.9 (24.0–60.8)	40.7 (27.5–59.0)	*n. s.*	36.8 (29.7–56.3)	*n. s.*
[Δ9-THC ng/ml/Δ9-THC-COOH ng/ml]	0.13 (0.06–0.17)	0.09 (0.04–0.14)	*n. s.*	0.14 (0.09–0.18)	*n. s.*

^a^*p* values were determined using the Mann-Whitney *U* test and are derived from the comparison of the respective variation with the wild type; if the *p* value is less than 0.05, the difference was considered statistically significant

*n. s.* not significant

## Discussion

The purpose of this study was to investigate the role of genetic variations in Δ9-THC pharmacokinetics in the analysis of DUID samples. Starting point was the knowledge of allele frequencies of CYP2C9 and CYP2C19 polymorphisms in Caucasians and their effect on the enzyme activity [18] on the one hand and on the other hand the study of Sachse-Seeboth et al. [21], which showed a threefold higher median area under the curve of Δ9-THC and a 70% lower one of Δ9-THC-COOH in CYP2C9*3/*3 homozygotes compared with CYP2C9*1/*1 homozygotes after oral administration of Δ9-THC. We assume when working with authentic DUID samples tested positive for cannabinoids, the most common form of application is the inhalation, which comes along with a rather small first-pass-effect, compared with the oral intake. Therefore, genetic variations can have a different effect between both forms of application. In addition, for DUID samples, neither the time of consumption nor the quantity consumed is usually known. For this reason, a large scattering within the plasma concentrations is to be expected. In the forensic-toxicological context, the mother substance/metabolite ratio is therefore often used to investigate possible influences of genetic variations. The results of this study showed significantly higher ratios of Δ9-THC/Δ9-THC-COOH for the carriers of the CYP2C9 variations CYP2C9*2 and CYP2C9*3 compared with the carriers of the corresponding wild-type alleles in DUID cases. Interestingly, despite unknown amount and timing of consumption, significantly lower values for plasma concentrations of Δ9-THC-COOH for carriers of the CYP2C9*3 variation were found, which is in line with the results of Sachse-Seeboth et al. [21] and the described decreased enzyme activity of CYP2C9*3 polymorphism [18]. It confirms the assumption that variants of the CYP2C9 enzyme influence both the metabolism of Δ9-THC and the formation of Δ9-THC-COOH. In the present study, the reduced metabolism of Δ9-THC could not be shown in higher Δ9-THC concentrations. This is probably due to the strong influence of the two variables’ consumption quantity and time of consumption, as well as the lack of the homozygous genotype CYP2C9*3/*3.

A clear differentiation between the single influences of the variations CYP2C9*2 and CYP2C9*3 is difficult on the basis of the investigated sample set because of the small group sizes. Nevertheless, the trend study suggests that enzyme activity is more reduced for variation *3 compared with variation *2. Moreover, homozygous genotype CYP2C9*3/*3 was not represented in the investigated sample set. Because of a gene dose effect, even lower Δ9-THC-COOH concentrations as well as a higher Δ9-THC/Δ9-THC-COOH ratio and most important higher concentrations of Δ9-THC can be expected for homozygote carriers. In literature, the effect of the variation CYP2C9*2 is not yet clarified. While in vitro studies showed a comparable decrease in enzyme activity for both variants of the CYP2C9 enzyme [19], no significant effect on the pharmacokinetics of cannabinoids could be detected by Sachse-Seeboth et al. for the CYP2C9*2 variant in vivo [21], in contrast to the present study. Thus, a study on a larger sample set is necessary to conclusively answer the question on the effect of the CYP2C9*2 variant.

For the variations of the CYP2C19 enzyme, no influence on the plasma concentrations investigated could be found. This can be explained by the small proportion of the CYP2C19 enzyme in the metabolism of Δ9-THC. In comparison with the CYP2C9 enzyme, the catalytic activity of the CYP2C19 enzyme for 11-hydroxylation is less than 2% [15].

Assuming that the genotype CYP2C9*3/*3 shows an even clearer influence on the metabolism of THC, various forensic questions regarding the interpretation of cannabinoid plasma concentrations must be discussed anew. Since we used the mother substance/metabolites ratio to investigate possible influences of genetic variations and were able to confirm these influences significantly, in the presence of a relevant polymorphism, we might have to question the use of this ratio in order to determine the last time of consumption [32]. It has to be shown whether the genetic influence is so extensive that the calculation of the last time of consumption is significantly affected and that this effect is so large in quantitative terms that it becomes relevant for the forensic assessment. The same applies for the interpretation of the plasma concentration of Δ9-THC-COOH. When assessing the last time of consumption and the impairment of performance, the consumption pattern is of decisive importance. Hereby, Δ9-THC-COOH is often used to differentiate between one-off/occasional and regular cannabis use [33]. In short, high Δ9-THC-COOH plasma concentrations indicate regular consumption due to cumulative effects. As shown in this study, the plasma concentration of Δ9-THC-COOH was significantly lower for carriers of the CYP2C9*3 variation compared with the wild-type. Therefore, a misinterpretation of Δ9-THC-COOH plasma concentrations resulting in occasional use instead of continuous use is possible.

## Conclusion

In summary, the results of the present study give additional data for the correlation of CYP2C9 gene variations with cannabinoid plasma concentrations in DUID cases. It shows that in the forensic interpretation of the analytical results, it might be necessary to include genetic variations for metabolizing enzymes because this can be a further cause for a great variability of the cannabinoid plasma concentrations or the metabolic ratio of Δ9-THC/Δ9-THC-COOH. If further research confirms that the metabolism of Δ9-THC and the formation of Δ9-THC-COOH are reduced in the presence of a CYP2C9 gene variant, those so-called poor metabolizer should be considered, for example, when applying the ratio of Δ9-THC/Δ9-THC-COOH to determine the last time of consumption. A controlled study with inhaled application form and the focus on the time-related detectability of cannabinoids in dependence of different consumption patterns must clarify whether and to what extent the CYP2C9 genotype must be taken into account in the assessment.

## Electronic supplementary material

Supplement 1 The investigated polymorphisms, their dbSNP number, primer pairs for amplification with the corresponding fragment length and the minisequencing primers. fwd = forward; rev = reverse (DOCX 24 kb)

Supplement 2 Chemical-toxicological and molecular genetic data of the toxicogenetic study (n.d. = not detected, < limit of detection); colouring highlights variations in the alleles (deep colour homozygous, faint colour heterozygous), wild types are uncoloured. (DOCX 39 kb)

Supplement 3 Demographic data of all subjects, divided into the CYP2C9 and CYP2C19 genotypes (DOCX 27 kb)
