# Supplementary material for: Toxicogenetic analysis of Δ9-THC-metabolizing enzymes
Source: Int J Legal Med. 2020 Jul 25;134(6):2095–103. doi: 10.1007/s00414-020-02380-3 (PMC7578149; doi:10.1007/s00414-020-02380-3)
Supplement: Supplementary file 1 — The investigated polymorphisms, their dbSNP number, primer pairs for amplification with the corresponding fragment length and the minisequencing primers. fwd = forward; rev = reverse (DOCX 24 kb) [file 414_2020_2380_MOESM1_ESM.docx]

**Supplement 1**

| SNP | dbSNP-number | PCR-Primer  (5‘-3‘) | Fragment-length | Minisequencing-Primer  (5’-3) |
| --- | --- | --- | --- | --- |
| 430 C>T CYP2C9*2 | rs1799853 | fwd:GGAGGATGGAAAACAGAGACTTAC rev:TAAGGTCAGTGATATGGAGTAGGG | 310 | rev:(T)_11_CGGGCTTCCTCTTGAACAC |
| 1075 A>C CYP2C9*3 | rs1057910 | fwd:ACCATCCTCTCTTTAAGTTTGCAT  rev:TGATACTATGAATTTGGGGACTTCG | 404 | fwd:(T)_15_TGCACGAGGTCCAGAGATAC |
| 681 G>A CYP2C19*2 | rs4244285 | fwd:AATTACAACCAGAGCTTGGCAT rev:AGCTTTTCCTATCCTGACATCCTTA | 519 | fwd:(T)_13_TTTTCCCACTATCATTGATTATTTCCC |
| 636 G>A CYP2C19*3 | rs4986893 | fwd:TAAAAGACAAATAGGCCGGGAATGT rev:TGTACTTCAGGGCTTGGTCAATA | 511 | rev:(T)_23_AAAAACTTGGCCTTACCTGGAT |
| -806 C>T CYP2C19*17 | rs12248560 | fwd:CCTACCTCCCATCCTCTATTAGATT rev:GGTCTTCTGATGCCCATCGT | 497 | fwd:(T)_23_TCAAATTTGTGTCTTCTGTTCTCAAAG |
