# Supplementary material for: Toxicogenetic analysis of Δ9-THC-metabolizing enzymes
Source: Int J Legal Med. 2020 Jul 25;134(6):2095–103. doi: 10.1007/s00414-020-02380-3 (PMC7578149; doi:10.1007/s00414-020-02380-3)
Supplement: Supplementary file 2 — Chemical-toxicological and molecular genetic data of the toxicogenetic study (n.d. = not detected, < limit of detection); colouring highlights variations in the alleles (deep colour homozygous, faint colour heterozygous), wild types are uncoloured. (DOCX 39 kb) [file 414_2020_2380_MOESM2_ESM.docx]

**Supplement 2**

| TG-No. | Δ9-THC | 11-OH-Δ9-THC | Δ9-THC-COOH | Δ9-THC / Δ9-THC-COOH | 2C9*2 | 2C9*3 | 2C19*2 | 2C19*3 | 2C19*17 |
| --- | --- | --- | --- | --- | --- | --- | --- | --- | --- |
|  | [ng/mL] | | | |  |  |  |  |  |
| 01-12 | 1.6 | 2.4 | 45.4 | 0.04 | C/C | A/C | G/G | G/G | C/C |
| 02-12 | 0.8 | n.d. | 27.5 | 0.03 | C/C | A/A | A/A | G/G | C/C |
| 05-12 | 2.2 | n.d. | 12.2 | 0.18 | C/T | A/C | G/G | G/G | C/C |
| 09-12 | 7.7 | 1.8 | 40.1 | 0.19 | C/T | A/A | G/G | G/G | C/T |
| 15-12 | 12.5 | 3.4 | 18.7 | 0.67 | C/T | A/C | G/G | G/G | C/C |
| 17-12 | 5.9 | 2.8 | 31.1 | 0.19 | C/C | A/C | G/G | G/G | C/C |
| 07-13 | 1.4 | n.d. | 18.9 | 0.07 | C/C | A/C | G/A | G/G | C/C |
| 08-13 | 5.1 | 2.7 | 39.3 | 0.13 | C/C | A/A | G/A | G/G | C/C |
| 10-13 | 6.9 | 3.5 | 82.5 | 0.08 | C/C | A/A | G/G | G/G | C/T |
| 11-13 | 4.0 | 1.7 | 8.6 | 0.47 | C/C | A/C | G/A | G/G | C/C |
| 12-13 | 1.7 | 0.4 | 12.5 | 0.14 | C/C | A/A | G/G | G/G | C/C |
| 13-13 | 5.4 | 2.2 | 39.3 | 0.14 | C/C | A/A | G/A | G/G | C/T |
| 14-13 | 5.1 | 2.1 | 18.9 | 0.27 | C/C | A/C | G/A | G/G | C/C |
| 15-13 | 1.7 | n.d. | 11.3 | 0.15 | C/C | A/C | G/G | G/G | C/T |
| 18-13 | 3.0 | 0.9 | 34.3 | 0.09 | C/C | A/A | G/G | G/G | C/T |
| 20-13 | 6.1 | 3.0 | 34.7 | 0.18 | C/C | A/A | G/G | G/G | T/T |
| 21-13 | 10.9 | 3.6 | 53.8 | 0.20 | C/C | A/A | A/A | G/G | C/C |
| 23-13 | 6.7 | 1.7 | 21.5 | 0.31 | C/C | A/A | G/G | G/G | T/T |
| 24-13 | 2.0 | 2.1 | 70.8 | 0.03 | C/C | A/A | G/G | G/G | C/C |
| 26-13 | 4.4 | 2.1 | 36.6 | 0.12 | C/C | A/A | G/G | G/G | C/C |
| 27-13 | 2.4 | 1.9 | 11.4 | 0.21 | C/C | A/C | G/G | G/G | C/T |
| 28-13 | 14.7 | 3.8 | 61.8 | 0.24 | C/C | A/A | G/G | G/G | C/T |
| 29-13 | 4.3 | 1.7 | 26.8 | 0.16 | C/C | A/A | G/G | G/G | C/C |
| 32-13 | 2.7 | n.d. | 17.8 | 0.15 | C/C | A/A | G/G | G/G | C/T |
| 33-13 | 5.9 | 2.2 | 73.2 | 0.08 | C/C | A/A | G/A | G/G | C/C |
| 35-13 | 3.4 | 1.8 | 40.7 | 0.08 | C/C | A/A | G/A | G/G | C/T |
| 38-13 | 7.7 | 1.6 | 51.2 | 0.15 | C/C | A/A | G/G | G/G | C/C |
| 40-13 | 1.7 | 1.2 | 53.1 | 0.03 | C/C | A/A | A/A | G/G | C/C |
| 41-13 | 5.2 | 2.8 | 59.0 | 0.09 | C/C | A/A | G/A | G/G | C/C |
| 42-13 | 0.9 | 0.9 | 22.0 | 0.04 | C/C | A/C | G/G | G/G | C/C |
| 43-13 | 10.6 | 2.1 | 51.7 | 0.21 | C/C | A/A | G/G | G/G | T/T |
| 44-13 | 0.7 | 0.5 | 15.8 | 0.04 | C/C | A/A | G/G | G/G | C/C |
| 45-13 | 1.1 | n.d. | 22.2 | 0.05 | C/T | A/A | G/G | G/G | C/T |
| 48-13 | 6.6 | 2.2 | 16.8 | 0.39 | C/C | A/A | G/G | G/G | C/C |
| 50-13 | 14 | 4.8 | 73.7 | 0.19 | C/C | A/A | G/G | G/G | C/C |
| 51-13 | 2.0 | 0.8 | 24.4 | 0.08 | C/C | A/A | G/G | G/G | C/C |
| 53-13 | 3.8 | 0.8 | 22.9 | 0.17 | C/T | A/A | G/G | G/G | C/T |
| 54-13 | 25 | 8.1 | 56.8 | 0.44 | C/C | A/A | G/G | G/G | C/C |
| 01-14 | 5.0 | 3.9 | 76.9 | 0.07 | C/C | A/A | G/G | G/G | C/C |
| 05-14 | 4.6 | n.d. | 81 | 0.06 | C/T | A/A | G/A | G/G | C/C |
| 06-14 | 2.3 | 0.7 | 15.5 | 0.15 | C/C | A/A | A/A | G/G | C/C |
| 07-14 | 16.8 | 5.7 | 41.6 | 0.40 | C/C | A/C | G/A | G/G | C/C |
| 08-14 | 6.4 | 2.3 | 21.5 | 0.30 | C/T | A/A | G/G | G/G | C/T |
| 09-14 | 3.6 | 1.7 | 9.2 | 0.39 | C/C | A/C | G/G | G/G | C/C |
| 11-14 | 16.7 | 4.1 | 60.4 | 0.28 | C/T | A/A | G/A | G/G | C/C |
| 13-14 | 3.1 | 1.7 | 36.8 | 0.08 | C/C | A/A | G/G | G/G | C/T |
| 16-14 | 8.5 | 5.7 | 52.3 | 0.16 | C/C | A/A | G/G | G/G | C/C |
| 17-14 | 5.4 | 2.3 | 27.6 | 0.20 | T/T | A/A | G/G | G/G | C/C |
| 19-14 | 1.7 | 1.1 | 20 | 0.09 | C/T | A/C | G/G | G/G | C/C |
| 20-14 | 1.0 | n.d. | 23.6 | 0.04 | C/C | A/A | G/A | G/G | C/C |
| 21-14 | 1.5 | n.d. | 22.6 | 0.07 | C/C | A/A | G/G | G/G | C/C |
| 22-14 | 7.9 | 2.1 | 56.3 | 0.14 | C/C | A/A | G/G | G/G | C/T |
| 24-14 | 5.2 | 1.5 | 12.2 | 0.43 | C/T | A/A | G/G | G/G | C/C |
| 25-14 | 10.6 | 2.0 | 33.1 | 0.32 | C/C | A/A | G/G | G/G | C/C |
| 26-14 | 1.4 | n.d. | 16.0 | 0.09 | C/C | A/A | A/A | G/G | C/C |
| 01-15 | 3.7 | 2.7 | 68.1 | 0.05 | C/C | A/A | G/G | G/G | C/C |
| 03-15 | 5.9 | 2.4 | 68.3 | 0.09 | C/C | A/A | G/G | G/G | C/T |
| 04-15 | 18 | 6.3 | 40.2 | 0.45 | C/T | A/C | G/G | G/G | C/C |
| 05-15 | 1.1 | 0.6 | 29.7 | 0.04 | C/C | A/A | G/G | G/G | C/T |
| 06-15 | 20.0 | 4.2 | 31.8 | 0.63 | C/C | A/A | G/G | G/G | C/T |
| 07-15 | 2.3 | 1.0 | 22.7 | 0.10 | C/C | A/A | G/G | G/G | C/T |
| 09-15 | 2 | 1.1 | 58.3 | 0.03 | C/C | A/A | G/G | G/G | C/C |
| 10-15 | 2.1 | 0.5 | 15.1 | 0.14 | C/C | A/A | G/G | G/G | C/T |
| 12-15 | 2.5 | 1.8 | 64.1 | 0.04 | C/C | A/A | G/A | G/G | C/C |
| 13-15 | 26.8 | 8.3 | 149 | 0.18 | C/C | A/A | G/A | G/G | C/T |
| 14-15 | 8.3 | 2.4 | 25.2 | 0.33 | C/T | A/A | G/G | G/G | C/C |
