# Supplementary material for: Toxicogenetic analysis of Δ9-THC-metabolizing enzymes
Source: Int J Legal Med. 2020 Jul 25;134(6):2095–103. doi: 10.1007/s00414-020-02380-3 (PMC7578149; doi:10.1007/s00414-020-02380-3)
Supplement: Supplementary file 3 — Demographic data of all subjects, divided into the CYP2C9 and CYP2C19 genotypes (DOCX 27 kb) [file 414_2020_2380_MOESM3_ESM.docx]

**Supplement 3**

|  | genotype | n | age^a^ | weight^a^ | length^a^ | BMI^b^ | nicotine^c^ consumption | alcohol^d^ consumption | cannabis^e^ consumption |
| --- | --- | --- | --- | --- | --- | --- | --- | --- | --- |
| CYP2C9 | ***1/*1** | 43 | 26  (6.4) | 82.0  (16.9) | 1.8  (0.1) | 24.8 (5.3) | NR = 3   R = 40 | 0 = 16  1 = 27 | x = 10 1 = 8  2 = 12  3 = 13 |
|  | ***1/*2** | 8 | 24  (2.4) | 72.9  (6.8) | 1.8  (0.1) | 21.9 (1.2) | NR = 0  R = 8 | 0 = 2 1 = 6 | x = 1 1 = 2 2 = 2 3 = 3 |
|  | ***2/*2** | 1 | 23 | 85.0 | 1.9 | 24.6 | NR = 0  R = 1 | 0 = 0 1 = 1 | x = 0 1 = 0 2 = 0 3 = 1 |
|  | ***1/*3** | 10 | 29  (9.0) | 78.6  (9.7) | 1.8  (0.1) | 23.7 (2.0) | NR = 1  R = 9 | 0 = 4 1 = 6 | x = 1 1 = 3 2 = 3 3 = 3 |
|  | ***2/*3** | 4 | 31  (6.3) | 76.5  (3.0) | 1.8  (0.0) | 23.2 (0.9) | NR = 0  R = 4 | 0 = 0 1 = 4 | x = 1  1 = 0 2 = 2 3 = 1 |
| CYP2C19 | ***1/*1** | 27 | 26  (7.3) | 81.6 (17.3) | 1.8 (0.0) | 24.4 (5.2) | NR = 2  R = 25 | 0 = 6  1 = 21 | x = 6 1 = 2 2 = 8  3 = 11 |
|  | ***1/*2** | 11 | 28 (6.1) | 76.8 (14.2) | 1.8 (0.1) | 24.0 (4.4) | NR = 0  R = 11 | 0 = 4 1 = 7 | x = 3 1 = 1 2 = 4 3 = 3 |
|  | ***2/*2** | 5 | 23 (3.4) | 80.2 (7.2) | 1.8 (0.0) | 24.5 (1.6) | NR = 0  R = 5 | 0 = 3 1 = 2 | x = 0 1 = 1 2 = 2 3 = 2 |
|  | ***1/*17** | 17 | 27 (6.9) | 79.9 (11.8) | 1.8 (0.1) | 24.0 (3.9) | NR = 1  R = 16 | 0 = 6  1 = 11 | x = 3 1 = 8 2 = 3 3 = 3 |
|  | ***2/*17** | 3 | 24 (5.1) | 73.0 (2.2) | 1.8 (0.1) | 23.3 (1.4) | NR = 2  R = 1 | 0 = 1 1 = 2 | x = 1  1 = 1 2 = 0 3 = 1 |
|  | ***17/*17** | 3 | 28 (4.2) | 87.3 (16.4) | 1.9 (0.1) | 24.9 (6.0) | NR = 0   R = 3 | 0 = 2 1 = 1 | x = 0 1 = 0 2 = 2 3 = 1 |

^a^ the mean values with the standard deviations are given

^b^ BMI = *body mass index* (body weight/body length^2^)

^c^ NR = non-smoker; R = smoker

^d^ 0 = no consumption; 1 = occasional/regular consumption

^e^ x = no information; 1= rare/one-time consumption; 2 = occasional consumption; 3 = regular consumption
